# Cervical Branch Retrograde Superficial Parotidectomy for Tail of Parotid Lesions

**DOI:** 10.1002/oto2.70053

**Published:** 2025-04-07

**Authors:** Chloe H. Amsterdam, Ryan T. Judd, Jeremy Godsell, Hilary C. McCrary, Janice L. Farlow, Enver Ozer

**Affiliations:** ^1^ The Ohio State University College of Medicine Columbus Ohio USA; ^2^ Department of Otolaryngology–Head and Neck Surgery The Ohio State University Columbus Ohio USA; ^3^ Department of Otolaryngology–Head and Neck Surgery University of Utah Salt Lake City Utah USA; ^4^ Department of Otolaryngology–Head and Neck Surgery Indiana University Indianapolis Indiana USA

**Keywords:** benign mass, facial nerve, head and neck surgery, parotidectomy, retrograde

## Abstract

Facial nerve dysfunction following superficial parotidectomy is one of the most well‐known and dreaded complications of the procedure, leading to significant postoperative impairments in affected patients. In lesions involving the parotid tail, the marginal mandibular branch is at particular risk. In contrast, injury to the cervical branch is usually of minimal consequence. Classically, facial nerve dissection in parotidectomy is performed anterograde from the main trunk. In patients presenting with benign superficial parotid tail lesions, however, we often begin with the identification of the cervical branch and perform retrograde dissection to decrease the risk of injury to both the main trunk and the marginal mandibular branch. This technique also allows for the preservation of the great auricular nerve, a shorter incision, and a smaller elevated facial flap, yielding better cosmetic and functional results without compromising the integrity of the resection. Here we describe this technique used for 5 consecutive patients with excellent outcomes.

Management of parotid lesions often involves superficial parotidectomy as the procedure can be both definitively diagnostic and curative.^1^ The facial nerve has a well‐known and intimate relationship with the parotid gland, and postoperative facial nerve dysfunction is a significant and feared risk of surgery.^2^ Damage to the nerve is an inherent risk to all variations of parotid surgery and can lead to transient or permanent facial disfigurement and functional impairment.

The inferior most projection of the parotid gland near the angle of the mandible and anterolateral to the sternocleidomastoid muscle is referred to as the “tail” of the gland and is a common location for salivary gland neoplasms. The marginal mandibular and cervical branches course through this region, making a limited dissection restricted to these branches ideal for lesions confined to the tail. While the marginal mandibular branch is of significant cosmetic and functional importance, injury to the cervical branch of the nerve, which innervates the platysma, is of relatively little consequence to most patients.

Retrograde facial nerve dissection is a method that can be employed during parotid surgery; however, it is less common and oftentimes more technically challenging due to the need for identification and dissection of thin, fragile distal nerve branches.^3^ In an effort to minimize the risk of nerve injury, we advocate utilizing retrograde dissection of the cervical branch for lesions confined to the tail of the parotid.

## Description of Technique

**Figure 1:**
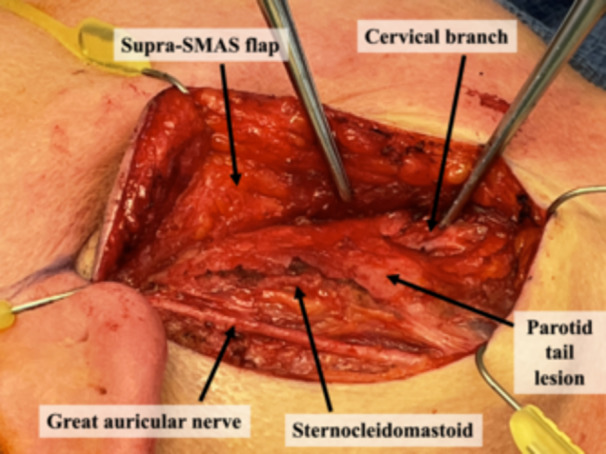
The major anatomical landmarks are the greater auricular nerve, sternocleidomastoid muscle, and the cervical branch of the facial nerve, which can be stimulated to demonstrate stimulation limited to the platysma muscle. SMAS, superficial musculoaponeurotic system.

**Figure 2:**
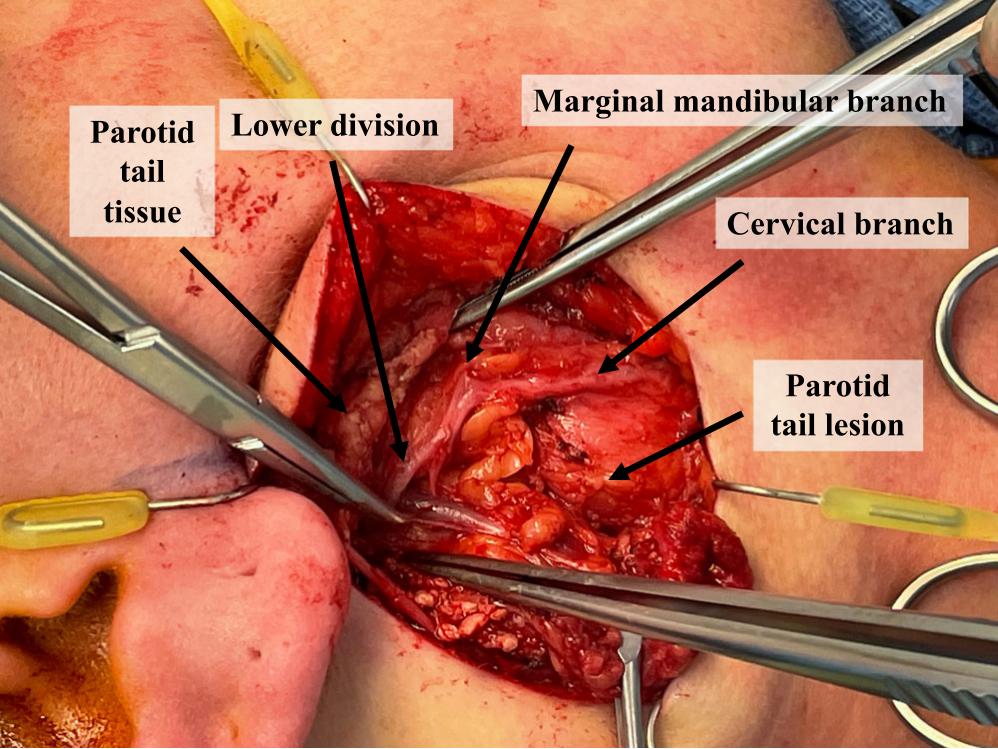
Here, the mass is further delineated. The lower division of the facial nerve is located with a clear distinction between the marginal mandibular nerve and the cervical branch of the facial nerve.

**Figure 3:**
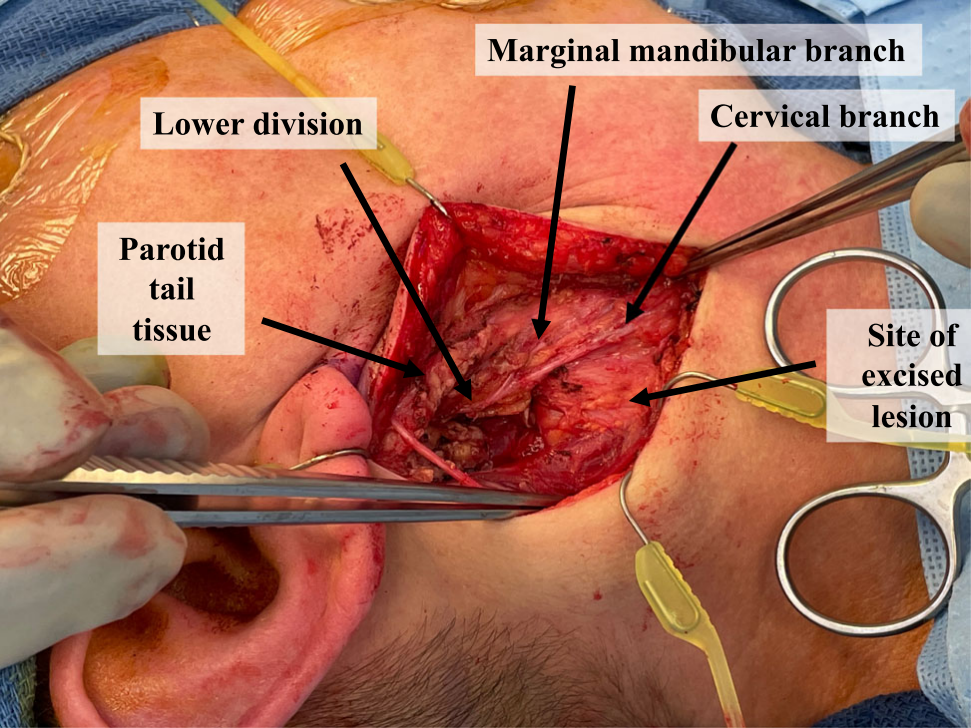
After removal, the surgeon can clearly appreciate the distinction between the marginal mandibular nerve versus the cervical branch of the facial nerve. Importantly, the course of the marginal mandibular nerve is completely preserved.

Patients appropriate for this technique must have the lesion limited to the tail of the parotid and suspected benign pathology on preoperative biopsy and imaging. An incision is designed underneath the lobule and extended into an existing crease in the neck; extension superiorly to the preauricular crease is not usually required. The incision is made, and a subplatysmal flap in the neck is elevated and extended to a supra‐superficial musculoaponeurotic system plane in the face. The great auricular nerve is identified traversing the sternocleidomastoid and preserved. The cervical branch of the facial nerve is identified in a consistent location 1 cm inferior to the angle of the mandible at the posterosuperior border of the submandibular gland (Figures 1 and 2).^4^ Confirmation of the nerve can be performed using stimulation and visualizing the movement of the platysma. This branch is traced proximally, and as it is delineated, the parotidomasseteric fascia is released from the sternocleidomastoid to free the inferolateral parotid. Retrograde dissection is continued along the cervical branch until the confluence of the cervical and marginal mandibular branches just posterior to the mandibular angle. The marginal mandibular nerve can then be traced anterograde, freeing it from the lesion. Further, retrograde dissection is typically not required for the tail of parotid lesions but can be performed if required (Figure 3). One closed suction drain is placed exiting posterior to the incision, and multilayered closure is performed.

## Results

This technique was performed on 5 consecutive patients with suspected benign tail of parotid lesions over a 6‐month period with the senior author as the attending surgeon. Patient demographic and pathologic characteristics are presented in Table 1. The main trunk of the facial nerve was not dissected in any case. The marginal mandibular nerve was traced to at least a small extent in 4 of 5 cases. All patients had full facial nerve function on postoperative day 1 and at 3‐week follow‐up based on clinical exam. All patients had benign pathology on the final pathology report.

**Table 1:** Patient Demographics and Pathology

Age	Sex	Pathology	Size, cm (largest dimension)	Marginal mandibular branch dissected?
34	M	Lipoma	5.2	Yes
56	M	Warthin's	4.0	Yes
50	F	Warthin's	4.5	Yes
81	M	Pleomorphic adenoma	3.3	Yes
73	F	Pleomorphic adenoma	1.5	No

Abbreviations: F, female; M, male.

## Discussion

In salivary gland lesions confined to the parotid tail, only the cervical and marginal mandibular nerve branches typically require dissection for safe resection of the mass, thus avoiding identification and possible injury to the main trunk of the nerve. The marginal mandibular branch is important functionally and cosmetically and is often thin and delicate making it prone to injury during distal identification and retrograde dissection. Alternatively, the cervical branch is found in a consistent location, can be more robust, and injury leads to often negligible morbidity, making it an ideal starting point for retrograde dissection.

Due to the inferior location of the tail of parotid lesions and the adjacent nerve branches, a smaller incision can be made entirely inferior to the auricle, offering improved cosmesis by avoiding an incision extending into the preauricular sulcus and disrupting the location of the lobule. The cervical branch retrograde approach also affords excellent visualization of the great auricular nerve (Figures 1 and 3). This allows the nerve to be spared and preserves sensation to the lobule, numbness of which is a common complaint following parotid surgery. In an anterograde approach, anterior branches of the great auricular nerve must often be sacrificed to safely identify the main trunk.

While retrograde parotidectomy is a well‐known option, there is little literature regarding this technique. Furusaka et al employed initial identification of the cervical branch; however, they then identified all branches of the facial nerve, rather than exclusively isolating the inferior 2 branches.^3^ Given our experience to date, this method is an excellent option for benign lesions in the tail of parotid.

## Conclusions

Resection of parotid lesions can be complicated by temporary or permanent facial nerve injury leading to substantial functional and aesthetic dysfunction. The anterograde technique involves dissection along the main trunk of the nerve, putting all distal branches at risk of postoperative paresis or paralysis. In benign lesions confined to the tail of the parotid, initial identification and subsequent retrograde dissection of the cervical branch is a reliable and low‐risk technique to avoid damage to the facial nerve.

## Author Contributions

**Chloe H. Amsterdam**, concept development, data analysis, writing, editing, image creation; **Ryan T. Judd**, concept development, data analysis, writing, editing, image creation; **Jeremy Godsell**, concept development, data analysis, writing, editing, image creation; **Hilary C. McCrary**, concept development, data analysis, writing, editing; **Janice L. Farlow**, data analysis, writing, editing; **Enver Ozer**, Concept development, data analysis, writing, editing.

## Disclosures

### Competing interests

None.

### Funding source

None.
